# Non-invasive assessment of esophageal and fundic varices in patients with primary biliary cholangitis

**DOI:** 10.1007/s00330-024-11049-z

**Published:** 2024-09-11

**Authors:** Yuan Zhang, Chunyang Huang, Fankun Meng, Xing Hu, Xiaojie Huang, Jing Chang, Xue Han, Tieying Zhang, Jing Han, Huiyu Ge

**Affiliations:** 1 Beijing Youan Hospital, Capital Medical University, No. 8, Xitoutiao, Youanmenwai, Fengtai District 100069 Beijing, China; 2 Beijing Chaoyang Hospital, Beijing, China

**Keywords:** Elastography, Esophageal and gastric varices, Spleen, Primary biliary cholangitis

## Abstract

**Objectives:**

The Baveno VII consensus recommends endoscopic screening for varicose veins in cases of liver stiffness measurement (LSM) ≥ 20 kPa or platelet count ≤ 150 × 10^9^/L. Whether this approach was appropriate for patients with primary biliary cholangitis (PBC) remains uncertain. This study expanded the observed risk factors by adding analysis of ultrasound images as a non-invasive tool to predict the risk of esophageal or fundic varices.

**Methods:**

We enrolled 111 patients with PBC whose complete ultrasound images, measurement data, and LSM data were available. The value of the periportal hypoechoic band (PHB), splenic area, and LSM in determining the risk of varicose veins and variceal rupture was analyzed. A prospective cohort of 67 patients provided external validation.

**Results:**

The area under the receiver operating characteristic curve (AUC) for predicting varicose veins using LSM > 12.1 kPa or splenic areas > 41.2 cm^2^ was 0.806 (95% confidence interval (CI): 0.720–0.875) and 0.852 (95% CI: 0.772–0.912), respectively. This finding could assist in avoiding endoscopic screening by 76.6% and 83.8%, respectively, with diagnostic accuracy surpassing that suggested by Baveno VII guidelines. The AUCs for predicting variceal rupture using splenic areas > 56.8 cm^2^ was 0.717 (95% CI: 0.623–0.798). The diagnostic accuracy of PHB for variceal rupture was higher than LSM and splenic areas (75.7% vs. 50.5% vs. 68.5%).

**Conclusion:**

We recommend LSM > 12.1 kPa as a cutoff value to predict the risk of varicosity presence in patients with PBC. Additionally, the splenic area demonstrated high accuracy and relevance for predicting varicose veins and variceal rupture, respectively. The method is simple and reproducible, allowing endoscopy to be safely avoided.

**Clinical relevance statement:**

The measurement of the splenic area and identification of the periportal hypoechoic band (PHB) on ultrasound demonstrated high accuracy and relevance for predicting the risk of esophageal or fundic varices presence and variceal rupture, respectively.

**Key Points:**

- *Predicting varices in patients with primary biliary cholangitis (PBC) can reduce the morbidity and mortality of gastrointestinal hemorrhage.*
- *Transient elastography (TE) and ultrasound play an important role in predicting patients with PBC with varices.*
- *TE and ultrasound can predict varicose veins and variceal rupture. Liver stiffness measurement and splenic area measurements can allow endoscopy to be safely avoided*.

## Introduction

Primary biliary cholangitis (PBC) is a chronic progressive cholestatic liver disease. Portal hypertension (PH) is a common complication of chronic progressive liver disease that can occur early in the course of PBC [1]. Although a mechanism has not been elucidated, several hypotheses for PH pathogenesis have been proposed, including granulomatous inflammation compressing the branches of the portal vein, leading to pre-sinusoidal PH, pre-sinusoidal fibrosis, and nodular regenerative hyperplasia [2]. PBC has an insidious onset but rapid progression. Variceal rupture and hemorrhage are among the most concerning complications of cirrhosis. The 3-year survival rate after variceal rupture and hemorrhage in patients with PBC is only 46% [3]; therefore, screening for PH to prevent gastrointestinal bleeding is of great clinical importance in these patients.

Esophagogastroduodenoscopy (EGD) can be used to identify patients at risk of variceal rupture, yet the prevalence of varices in patients with compensated cirrhosis is only 5–15% in actual clinical practice [4]. A major problem is avoiding unnecessary endoscopic screening and non-invasively predicting the risk of variceal rupture due to PH. Transient elastography (TE) is a non-invasive technique that can be used to accurately determine liver fibrosis by measuring liver stiffness. Growing evidence suggests that TE can reflect the hepatic venous pressure gradient (HVPG) for PH assessment [5]. The guidelines of the Baveno VII Consensus Workshop recommended that EGD screening could be performed in patients with compensated cirrhosis, liver stiffness measurement (LSM) ≥ 20 kPa, or platelet count ≤ 150 × 10^9^/L [6]. Studies have also questioned the value of TE in predicting clinically significant PH (CSPH), suggesting that it is not sufficiently accurate to replace HVPG [7, 8]. The spleen enlarges with disease progression in most patients with PBC, with characteristic features on ultrasound [9]. Thus, the present study investigated the effectiveness of TE and ultrasound imaging in predicting PBC in patients with esophageal or fundic varices.

## Material and methods

### Patients

**Fig. 1:**
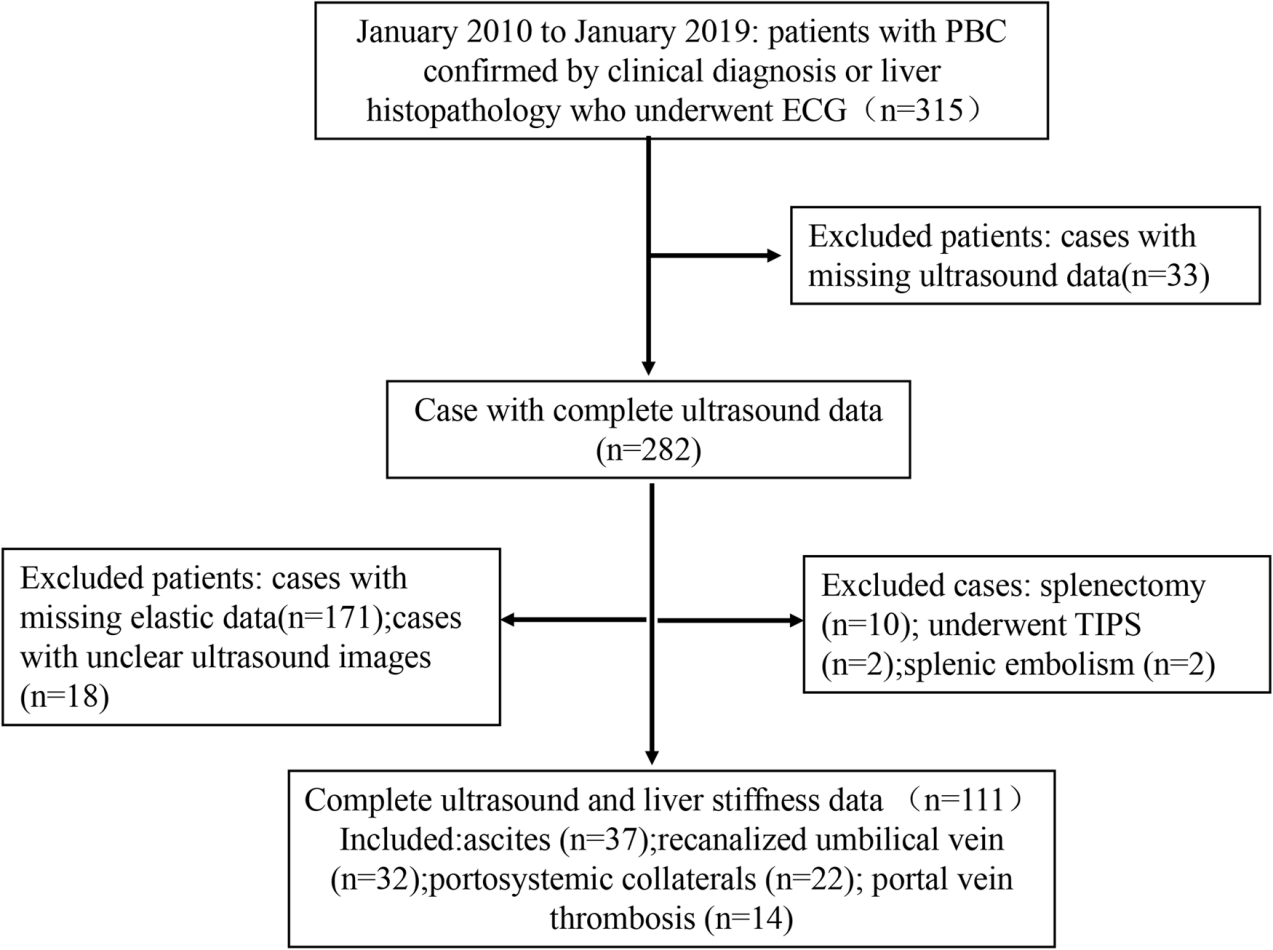
Study flowchart

Data from patients with PBC admitted to Beijing Youan Hospital between January 2010 and January 2019 were retrospectively analyzed. The study included 315 patients with PBC who showed endoscopic findings. Diagnosis of PBC was based on the results of liver function tests, the presence of serum antimitochondrial antibodies, or histopathologic findings. Of the 315 patients with complete laboratory findings, 282 had complete ultrasound data available (Fig. 1). The prospective cohort served as a validation cohort and included 85 patients with PBC with endoscopic findings between April 2019 and March 2023. This study was conducted in accordance with the principles of the Declaration of Helsinki and Istanbul and was approved by the Institutional Review Board of Beijing Youan Hospital: 2010–2019 (retrospective study) and 2019–2022 (prospective study). The requirement for informed consent was waived for the retrospective cases and informed consent was obtained for the prospective cases.

### Data extraction

Patient demographics were collected from a database query of the Beijing Youan Hospital electronic medical record system. Ultrasound reports of patients were searched before or 2 months after endoscopy to determine LSM, portal vein internal diameter, splenic vein internal diameter, and spleen length and thickness (the splenic area was thus derived using the formula: 0.8 × spleen thickness × spleen length), and ultrasound images of the liver were observed to identify ultrasound manifestations of periportal hypoechoic band (PHB) [9]. The images were assessed by two sonographers in parallel double-blind judgment, both with more than 10 years of experience [9]. Discrepancies in subjective observations were resolved through discussion before reaching a unanimous judgment. All ultrasound manifestations associated with PH, including ascites, recanalized umbilical veins, varices below the left lobe of the liver or at the splenic hilum, and portal vein thrombosis, were documented.

Diagnosis of gastroesophageal varices was made based on upper gastrointestinal endoscopy findings according to the simplified classification system proposed in the UK guidelines on the management of variceal hemorrhage in patients with cirrhosis [10]. Grade 0: No varicose veins; Grade 1: varices that collapse to inflation of the esophagus with air; Grade 2: varices between grades 1 and 3; Grade 3: varices that are large enough to occlude the lumen. The patients were classified into hemorrhage group (F1) and non-hemorrhage (F0) group based on the presence or previous occurrence of gastrointestinal hemorrhage at the time of treatment, and into non-varices (C1) and varices (C2) groups based on the presence or absence of esophageal or fundic varices.

Laboratory tests were performed within two months before or after EGD, including alanine aminotransferase, aspartate aminotransferase, total bilirubin, r-glutamyltransferase, alkaline phosphatase, albumin, and platelet count (PLT).

### Statistical analysis

The statistical analysis was performed using IBM SPSS Statistics for Windows, version 26.0, MedCalc (version 15.2.2), and GraphPad Prism (8.0.2) software. Measurements with normal distributions are expressed as means ± standard deviation and intergroup comparisons were performed using the *t*-test. Measurements with non-normal distributions are expressed as medians (minimum, maximum), and intergroup comparisons were performed using the Mann–Whitney *U*-test. Count data were expressed as *n* (%), and intergroup comparisons were performed using the *X*^2^ test. Independent risk factors for the development of varices in patients with PBC and for variceal rupture and hemorrhage were analyzed by logistic regression. The diagnostic efficacies of risk factors for predicting the development of esophageal and fundic varices as well as variceal rupture and hemorrhage were analyzed using the receiver operating characteristic (ROC) curve, and the optimal diagnostic thresholds for each factor were calculated using Youden’s index. Differences with *p* < 0.05 were considered statistically significant.

## Results

### General characteristics

In this study, 282 patients with PBC underwent EGD, 10 underwent splenectomy, two underwent TIPS, and two underwent splenic embolization. Esophageal or fundic varices were present in 217 patients (77.0%). Patients in the varices group were older (60 years vs. 50 years, *p* < 0.001) and had lower PLT (91 × 10^9^/L vs. 196 × 10^9^/L, *p* < 0.001). Symptoms of upper gastrointestinal hemorrhage were present in 56 cases either previously or at the time of examination. Additionally, 85 patients (30.1%) presented with ultrasound manifestations associated with PH during the study period. Furthermore, 37 patients developed ascites, 32 developed a recanalized umbilical vein, 22 developed portosystemic collaterals, and 14 developed portal vein thrombosis. The demographic characteristics of the 111 patients with PBC ultimately included in the study are detailed in Table 1 and Fig. 1.

**Table 1:** Baseline demographic and clinical characteristics of patients

Variable	Retrospective cohort (*n* = 112)	External cohort (*n* = 67)
Age, years	56 (37–83)	59 (34–77)
Sex (female)	100 (89.3%)	57 (85.1%)
ALT, U/L	47.9 (4.5–447.6)	22.9 (9.0–574.1)
AST, U/L	62.0 (15.8–812.3)	43.4 (16.9–259.5)
TBIL, μmol/L	22.6 (8.4–467.8)	23.6 (6.0–381.8)
GGT, U/L	188.3 (15.5–1837.3)	73.2 (1.1–1742.1)
ALP, U/L	197.0 (41.7–1094.8)	159.0 (62.0–1100.4)
ALB, g/L	37.8 (20.9–48.6)	38.5 (23.3–85.0)
PLT, ×10^9^/L	113 (15–368)	96 (25–298)
Esophageal varices grading		
Grading 0	39 (34.8%)	23 (34.3%)
Grading 1	24 (21.4%)	22 (32.8%)
Grading 2	7 (6.2%)	12 (17.9%)
Grading 3	42 (37.5%)	10 (14.9%)
Gastric varices grading		
Grading 0	77 (68.8%)	53 (79.1%)
Grading 1	33 (29.5%)	14 (20.9%)
Grading 2	1 (0.9%)	0 (0%)
Grading 3	1 (0.9%)	0 (0%)
LSM, kPa	20.9 (4.4–75)	17.5 (4.7–75.0)
Abodominal ultrasound		
Portal vein internal diameter, mm	12 (9–19)	12 (5–20)
Splenic area, cm^2^	50.2 (19.3–138)	48.6 (13.8–119.9)
Splenic vein internal diameter, mm	8 (6–13)	8 (6–14)
PHB	63 (56.3%)	40 (59.7%)

Data are shown as median (range)

*ALT* alanine aminotransferase, *AST* aspartate aminotransferase, *TBIL* total bilirubin, *GGT* r-glutamyltransferase, *ALP* alkaline phosphatase, *ALB* albumin, *PLT* platelet count, *LSM* liver stiffness measurements, *PV* portal vein internal diameter, *SV* splenic vein internal diameter, *PHB* periportal hypoechoic band

In this study, 93% (264/282) of patients had clear liver ultrasound images on which PHB was accurately identified, 41% (116/282) underwent TE testing, and five patients who underwent splenectomy were excluded, leaving 111 patients with complete ultrasound and TE data who were included in the study.

### Stratified analysis of varices

**Fig. 2:**
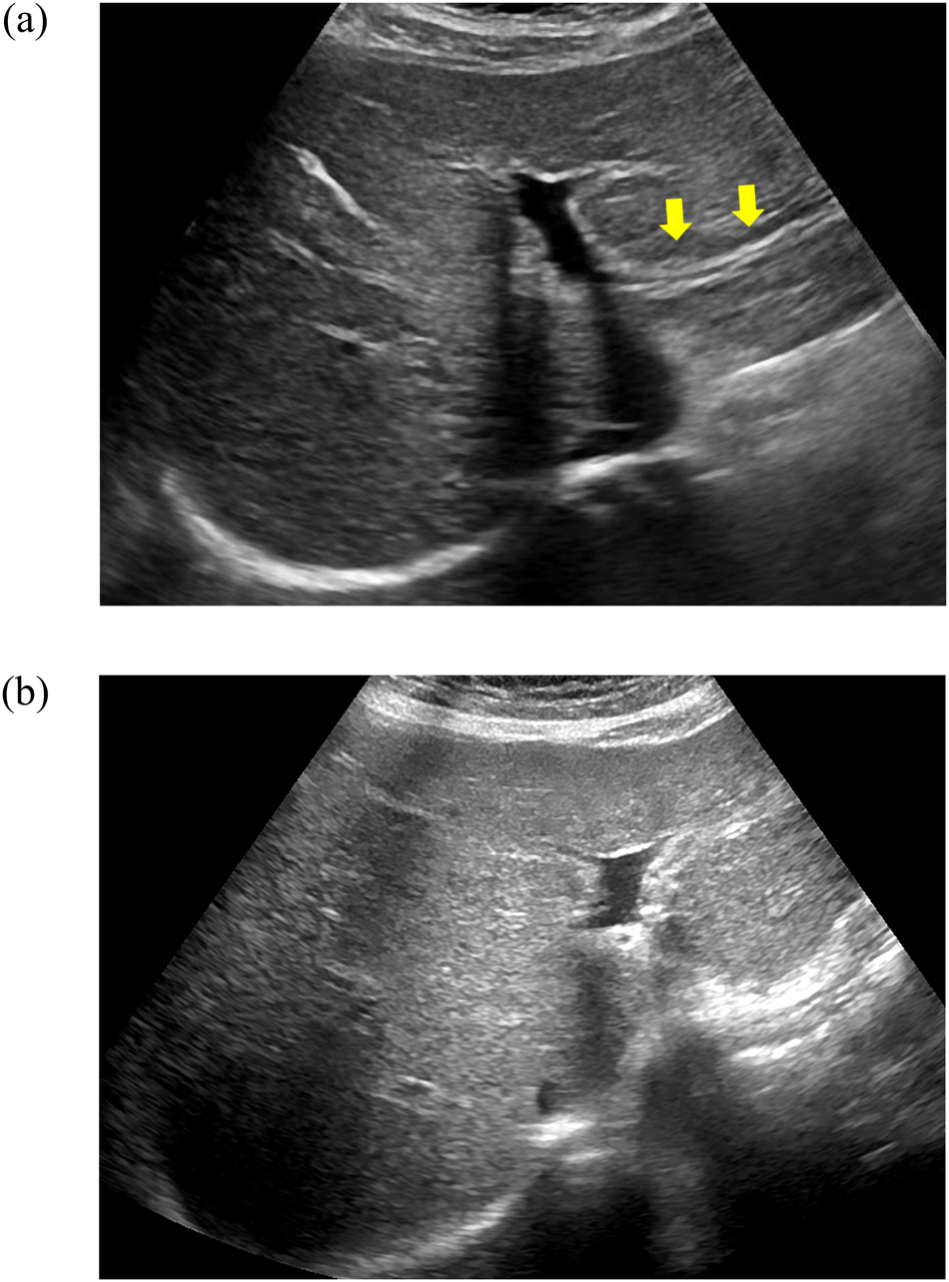
With and without PHB around the portal vein on ultrasound. **a** Hypoechoic band can be seen around the portal vein. **b** Hypoechoic band can not be seen around the portal vein. PHB, periportal hypoechoic band

The relevant measurements and LSM suggestive of manifestations of PH, including portal vein internal diameter (12 mm vs. 11 mm), splenic area (61.7 cm^2^ vs. 37.4 cm^2^), splenic vein internal diameter (9 mm vs. 7 mm), and LSM (24.4 kPa vs. 10.3 kPa), were higher in the varices group (C2) than in the non-varices group (C1) (*p* < 0.001). The incidence of PHB in the esophageal or fundic varices subgroup increased with increasing severity (Table 2). Patients with PBC with PHB (Fig. 2a) on ultrasound had significantly lower PLT (92.5 × 10^9^/L vs. 155 × 10^9^/L, *p* < 0.001) counts and higher splenic area (59.7 cm^2^ vs. 42.3 cm^2^, *p* < 0.001) than the group without PHB (Fig. 2b).

**Table 2:** Characteristics of patients with LSM and ultrasonic measurement parameters in different grading of esophageal varices or gastric varices

	PHB (%)	Splenic area (cm^2^)	PV (mm)	SV (mm)	LSM (kPa)
Esophageal varices grading					
Grading 0	51.3% (20/39)	37.7 (19.3–104.9)	11 (9–13)	7 (6–11)	10.4 (4.4–75)
Grading 1	66.7% (16/24)	58.9 (30–93.7)	11.5 (9–14)	8.5 (6–13)	27.5 (5.4–75)
Grading 2	28.6% (2/7)	50.2 (34–81.2)	11 (9–14)	8 (7–10)	27.3 (8–61.6)
Grading 3	82.9% (34/41)	67 (26.9–138)	12 (11–19)	9 (6–13)	22.3 (9.7–75)
Gastric varices grading					
Grading 0	45.5 (35/77)	44.2 (19.3–93.7)	11 (9–19)	8 (6–12)	16.5 (4.4–75)
Grading 1	78.1 (25/32)	69.0 (30–138)	12 (10–17)	9 (6–13)	23.8 (10.2–75)
Grading 2	100 (1/1)	47.5 (47.5–47.5)	12 (12–12)	7 (7–7)	72 (72–72)
Grading 3	100 (1/1)	43 (43–43)	12 (12–12)	6 (6–6)	11.2 (11.2–11.2)

*PHB* periportal hypoechoic band, *PV* portal vein internal diameter, *SV* splenic vein internal diameter, *LSM* liver stiffness measurements

The area under the ROC curve (AUC) values for the diagnosis of varices using LSM, splenic area, and PHB alone were 0.806 (95% confidence interval (CI): 0.720–0.875), 0.852 (95% CI: 0.772–0.912), and 0.736 (95% CI: 0.644–0.816), respectively. The optimal diagnostic thresholds for LSM and splenic area were > 12.1 kPa and > 41.2 cm^2^, respectively. The application of the three methods could avoid 76.6%, 83.8%, and 73.0% of endoscopy, respectively.

**Fig. 3:**
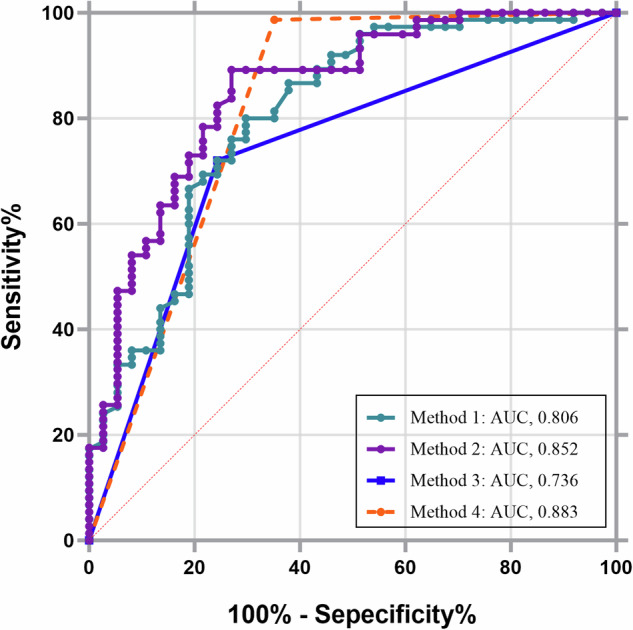
ROC curves for four methods for the diagnosis of varicose veins. Method 1: LSM; method 2: splenic area; method 3: PHB; method 4: Baveno VII guidelines. LSM, liver stiffness measurements; PHB, periportal hypoechoic band

Based on the Baveno VII consensus, using LSM ≥ 20 kPa or PLT ≤ 150 × 10^9^/L as diagnostic thresholds resulted in an AUC of 0.883. This showed an increase in the rates of diagnostic accuracy by 11.7% (13/111), 4.5% (5/111), and 15.3% (17/111) compared with the diagnostic thresholds of LSM > 12.1 kPa, splenic area > 41.2 cm^2^, and PHB, respectively. This also demonstrated the relatively high accuracy of a threshold splenic area of > 41.2 cm^2^ and Baveno VII consensus for predicting varices, whereas there was no significant difference in diagnostic efficacy (*p* = 0.4517) (Table 3 and Fig. 3).

**Table 3:** Diagnostic test characteristics of different methods for the diagnosis of varicose veins

	Cutoff	AUC	Se, %	Sp, %	PPV (%)	NPV (%)	LR +	LR −	Avoid endoscopy(%)
Method 1: LSM	12.1	0.806	79.7	70.3	84.3	63.4	2.68	0.29	76.6%
Method 2: Splenic area	41.2	0.852	89.2	73	86.8	77.1	3.3	0.15	83.8%
Method 3: PHB	—	0.736	71.6	75.7	—	33.3	—	1	73.0%
Method 4: Baveno VII guidelines	LSM ≥ 20 kPa or PLT ≤ 150 * 10^9^/L	0.818	98.7	64.9	85.1	96.0	2.81	0.021	88.3%

*LSM* liver stiffness measurements, *PHB* periportal hypoechoic band, *AUC*, area under the receiver operating characteristic curve, *Se* sensitivity, *Sp* specificity, *PPV* positive predictive value, *NPV* negative predictive value, *LR+* positive likelihood ratios, *LR*− negative likelihood ratios

### Predictors of high risk of variceal rupture

In the study cohort, 16.2% (18/111) of patients with PBC experienced rupture and hemorrhage of esophageal or fundic varices. The incidence of PHB was higher in the hemorrhage group (F1) than in the non-hemorrhage group (F0) (88.9% vs. 49.5%, *p* < 0.001), with grade 3 esophageal varices accounting for 88.9% of the patients (16/18) and grade 3 gastric varices for 0%, indicating that esophageal varices were more likely to lead to rupture and hemorrhage. The risk factors associated with variceal rupture and hemorrhage included age, LSM, splenic area, PHB, alanine aminotransferase, white blood cell count, and PLT. After adjusting for potential confounders, only PHB was strongly associated with the development of esophageal or fundic variceal rupture and hemorrhage (odds ratio (OR) = 8.174, 95% CI: 1.779–37.566, *p* = 0.007) (Table 4).

**Table 4:** Univariate and multivariate regression analyses of the risk in variceal rupture

	Univariate analysis		Multivariable analysis	
	OR (95% CI)	*p*-value	OR (95% CI)	*p-*value
Variable				
Age, years	1.054 (0.999–1.112)	0.052	—	—
LSM, kPa	1.013 (0.988–1.037)	0.313	—	—
Splenic area, mm^2^	1.031 (1.009–1.053)	**0.005**	—	—
PHB, mm	8.174 (1.779–37.566)	**0.007**	8.174 (1.779–37.566)	**0.007**
ALT, U/L	0.974 (0.953–0.995)	**0.017**	—	—
PLT, × 10^9^/L	0.988 (0.978–0.998)	**0.023**	—	—

Bold values mean *p* < 0.05

*LSM* liver stiffness measurements, *PHB* periportal hypoechoic band, *ALT* alanine aminotransferase, *PLT* platelet count

**Fig. 4:**
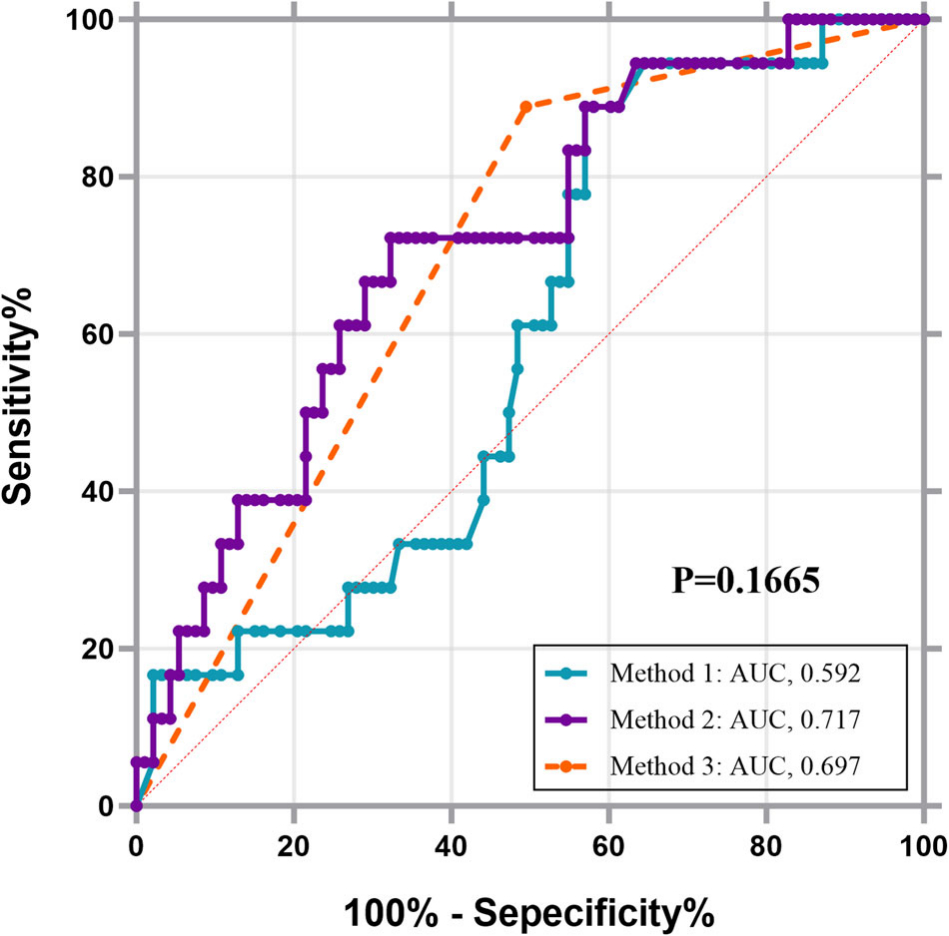
ROC curves for three methods for the diagnosis of variceal rupture. Method 1: LSM; method 2: splenic area; method 3: PHB. LSM, liver stiffness measurements; PHB, periportal hypoechoic band

The use of LSM for determining variceal rupture and hemorrhage (Method 1) demonstrated an AUC of 0.592 and a diagnostic threshold of > 12.2 kPa, a sensitivity of 0.889, and a specificity of 0.430, indicating that LSM alone had a low diagnostic efficacy for determining the presence or absence of upper gastrointestinal hemorrhage. The use of the splenic area alone (Method 2) for determining gastrointestinal hemorrhage had an AUC of 0.717 and a diagnostic threshold of > 56.8 cm^2^. The use of PHB (Method 3) for determining upper gastrointestinal hemorrhage had a sensitivity of 0.889, a specificity of 0.505, and an AUC of 0.720. The diagnostic efficacies did not differ significantly among the three methods (*p* = 0.1665–0.7788). The diagnostic accuracies of the three methods were 50.5%, 68.5%, and 75.7% respectively (Table 5 and Fig. 4).

**Table 5:** Diagnostic test characteristics of different methods for the diagnosis of variceal rupture

	Cutoff	AUC	Se, %	Sp, %	PPV (%)	NPV (%)	LR +	LR −	Avoid endoscopy(%)
Method 1: LSM	12.2	0.592	88.9	43	23.2	95.2	1.56	0.26	50.5%
Method 2: Splenic area	56.8	0.717	72.2	67.7	30.2	92.6	2.24	0.41	68.5%
Method 3: PHB	—	0.697	88.9	50.5	—	83.8	—	1	56.8%

*LSM* liver stiffness measurements, *PHB* periportal hypoechoic band, *AUC* area under the receiver operating characteristic curve, *Se* sensitivity, *Sp* specificity, *PPV* positive predictive value, *NPV* negative predictive value, *LR+* positive likelihood ratios, *LR*− negative likelihood ratios

### Validation of models based on new methods using the external validation cohort

The general and demographic characteristics of the 67 patients prospectively analyzed as an external validation cohort are shown in Table 1. The prevalence of esophageal or fundic varices was 82.1%. The AUC values for using the splenic area to predict varices and rupture and hemorrhage were 0.735 and 0.616, respectively. Endoscopic screening could be avoided in 76.1% of cases and correctly predict the risk of variceal rupture in 62.7% of patients with PBC.

## Discussion

Screening for varices in patients with PBC and PH can effectively reduce the morbidity and mortality of gastrointestinal hemorrhage. However, the prediction of rupture or hemorrhage from esophageal and fundic varices remains challenging. Current research is focused on the use of non-invasive tests to replace HVPG measurement and reduce unnecessary endoscopic screening. In the present study, we used multiple non-invasive methods to predict varices needing treatment while investigating the applicability of the Baveno VII consensus guidelines to patients with PBC, aiming to complement non-invasive predictive tools for PH.

PH with HVPG ≥ 10 mmHg is associated with the development of esophageal varices and poor prognosis [8]. HVPG measurement is currently recommended in international studies as a diagnostic indicator for the accurate determination of CSPH [11]. However, HVPG is a moderately invasive procedure and most patients with chronic liver disease require long-term follow-up. TE is a common non-invasive clinical examination that plays an important role in the determination of CSPH. Previous findings suggest that the risk of CSPH may be very low (< 9%) for LSM < 13.6 kPa [12]. A meta-analysis by the American Gastroenterological Association Institute using a range of LSM cutoffs (14.6–47.2 kPa) reported in 15 studies for the detection of high-risk EV indicated that the recommended cutoff of ≤ 19.5 (±2) kPa may misclassify 0.6% patients as not having high-risk EVs and 41.8% patients as having high-risk EVs due to the wide range of cutoffs [13]. Augustin et al concluded that CSPH can be accurately determined using an LSM cutoff of > 25 kPa and that the positive predictive power is slightly reduced with an LSM cutoff of > 21.1 kPa, although this reduced the proportion of patients in the LSM gray area [14]. In the present study, predicting esophageal or fundic varices using an LSM cutoff of > 12.1 kPa allowed endoscopic screening to be avoided in 77% of patients, with a false-negative rate of 20.3% and a false-positive rate of 29.7%.

Based on the Baveno VII guidelines, endoscopic screening can be avoided for a subset of patients who have undergone follow-up with repeated TE examinations and PLT, whereas patients with LSM ≥ 20 kPa or PLT ≤ 150 × 10^9^/L should undergo endoscopy for varices screening [6]. However, only using a diagnostic threshold of LSM ≥ 20 kPa, the diagnostic accuracy in the present cohort of patients with PBC was lower compared with a diagnostic threshold of LSM > 12.1 kPa, and the rate of misdiagnosis increased by 5.4%. LSM ≥ 21 kPa has been used as a threshold for the clinical prediction of CSPH in hepatitis C [15]. The diagnostic threshold for alcoholic cirrhosis is higher than that for viral cirrhosis (34.9 kPa vs. 20.5 kPa) [12]. Thus, the LSM threshold for patients with PBC is lower than those for other chronic liver diseases, demonstrating that PH is already present in the early stages of PBC, consistent with the findings reported by Murata et al One possible mechanism for PH development in the early stages of PBC is related to granulomatous inflammation compressing the branches of the portal vein, leading to pre-sinusoidal PH, pre-sinusoidal fibrosis, and nodular regenerative hyperplasia [16]. These results indicated that when LSM was the only reference value, the diagnostic accuracy for varicose veins with LSM ≥ 20 kPa was slightly lower than that in our study. Instead, we recommend that LSM > 12.1 kPa may be a manifestation of PH and suggest that patients with PBC undergo endoscopy to screen for varices. Although recommended as a non-invasive means of reducing endoscopic screening, vibration-controlled TE is not intended as an alternative to EGD [13].

The umbilical vein is the most specific sign of PH on ultrasound, with a characteristic sign being the appearance of dilated veins in the falciform ligament [17]. Similarly, ascites and portosystemic collaterals are ultrasound manifestations suggestive of PH. However, not all patients with PH present with these manifestations; in the present study, 38.2% of patients (42/110, with one patient who underwent splenectomy and one patient who underwent splenoembolization) presenting with varices did not present with ultrasound manifestations. The periportal halo or PHB is a characteristic imaging feature in patients with progressive PBC and is believed to be associated with periportal fibrosis or inflammatory cell infiltration. In PBC, the PHB gradually widens with disease progression [9]. In the present study, the incidence of varices was significantly increased when PHB was present, and PLT was significantly lower than in patients without PHB. The incidence rates of esophageal varices and fundic varices were 84.1% and 44.4%, respectively, in the PHB group and 40.8% and 14.3%, respectively, in the non-PHB group. The incidence of PHB was higher in the PH rupture and hemorrhage group than in the non-hemorrhage group, suggesting that the presence of PHB is predictive of disease progression in patients with PBC. In addition, splenomegaly is the most common feature of CSPH [18]. The splenic area plays a role in the prediction of esophageal or fundic varices and the risk of variceal rupture and hemorrhage, with a predictive power not inferior to that of LSM. This is primarily due to its simplicity of calculation, the lack of constraints, and its objective nature. Thus, the measurement of splenic area may be a promising alternative at institutions where TE is not available. We recommend the risk stratification of patients with PBC based on splenic area. In patients with splenic area ≤ 41.2 cm^2^, regular follow-up is recommended, and endoscopic screening is not needed. When the splenic area is 41.2–56.8 cm^2^, varices are likely present, and endoscopic screening is highly recommended. Finally, when the splenic area is > 56.8 cm^2^, the risk of variceal rupture and hemorrhage is high, and clinicians should implement appropriate measures to prevent gastrointestinal hemorrhage.

This study has several limitations. First, due to partially missing data, an insufficient number of retrospective cases were included for analysis. Second, due to the retrospective nature of the study, only subjective judgment could be made on PHB using static ultrasound images, which cannot fully reflect the prediction of PHB for varicose veins risk. In a future study, we will further standardize the storage of PHB images, increase the storage of dynamic images, and record the existence of PHB ultrasonic findings. In addition, our analysis of PHB should not only be limited to the analysis of images, it should have further increased the quantitative measurement. Finally, we did not analyze the long-term survival of patients with PBC evaluated with the two techniques; this assessment will be the focus of future studies.

In conclusion, the results of the present study suggested LSM > 12.1 kPa as a risk threshold value for predicting the presence of esophageal or fundic varices in patients with PBC. The splenic area showed high accuracy and relevance for predicting varicose veins and variceal rupture and can be used for prediction at thresholds of > 41.2 cm^2^ and > 56.8 cm^2^ respectively. LSM and splenic area measurements are simple and highly reproducible and can allow endoscopy to be safely avoided.

## Abbreviations

AUC: Area under the receiver operating characteristic curve
CI: Confidence interval
CSPH: Clinically significant portal hypertension
EGD: Esophagogastroduodenoscopy
HVPG: Hepatic venous pressure gradient
LSM: Liver stiffness measurement
OR: Odds ratio
PBC: Primary biliary cholangitis
PH: Portal hypertension
PHB: Periportal hypoechoic band
PLT: Platelet count
ROC: Receiver operating characteristic
TE: Transient elastography

## References

[1] Takeshita E, Matsui H, Shibata N et al (2004) Earlier recurrence of esophageal varices, following therapy, in patients with primary biliary cirrhosis (PBC) compared with non-PBC patients. J Gastroenterol 39:1085–1089 15580402 10.1007/s00535-004-1447-1

[2] Nakanuma Y, Ohta G (1987) Nodular hyperplasia of the liver in primary biliary cirrhosis of early histological stages. Am J Gastroenterol 82:8–10 3799585

[3] Imam MH, Lindor KD (2014) The natural history of primary biliary cirrhosis. Semin Liver Dis 34:329–333 25057955 10.1055/s-0034-1383731

[4] D’Amico G, Pasta L, Morabito A et al (2014) Competing risks and prognostic stages of cirrhosis: a 25-year inception cohort study of 494 patients. Aliment Pharmacol Ther 39:1180–1193 24654740 10.1111/apt.12721

[5] Kim MY, Jeong WK, Baik SK (2014) Invasive and non-invasive diagnosis of cirrhosis and portal hypertension. World J Gastroenterol 20:4300–4315 24764667 10.3748/wjg.v20.i15.4300 PMC3989965

[6] de Franchis R, Bosch J, Garcia-Tsao G, Reiberger T, Ripoll C, Baveno VII Faculty (2022) Baveno VII- renewing consensus in portal hypertension. J Hepatol 76:959–974 35120736 10.1016/j.jhep.2021.12.022 PMC11090185

[7] Llop E, Berzigotti A, Reig M et al (2012) Assessment of portal hypertension by transient elastography in patients with compensated cirrhosis and potentially resectable liver tumors. J Hepatol 56:103–108 21827733 10.1016/j.jhep.2011.06.027

[8] Kim G, Kim MY, Baik SK (2017) Transient elastography versus hepatic venous pressure gradient for diagnosing portal hypertension: a systematic review and meta-analysis. Clin Mol Hepatol 23:34–41 28263953 10.3350/cmh.2016.0059 PMC5381827

[9] Zhang Y, Hu X, Chang J et al (2023) Ultrasound imaging findings in primary biliary cholangitis. BMC Gastroenterol 23:448 38114916 10.1186/s12876-023-03083-w PMC10729522

[10] Jalan R, Hayes PC (2000) UK guidelines on the management of variceal haemorrhage in cirrhotic patients. British Society of Gastroenterology. Gut 46:III1–III15 10862604 10.1136/gut.46.suppl_3.iii1 PMC1766736

[11] de Franchis R, Faculty BVI (2015) Expanding consensus in portal hypertension: Report of the Baveno VI Consensus Workshop: stratifying risk and individualizing care for portal hypertension. J Hepatol 63:743–752 26047908 10.1016/j.jhep.2015.05.022

[12] Castera L, Pinzani M, Bosch J (2012) Non invasive evaluation of portal hypertension using transient elastography. J Hepatol 56:696–703 21767510 10.1016/j.jhep.2011.07.005

[13] Singh S, Muir AJ, Dieterich DT, Falck-Ytter YT (2017) American Gastroenterological Association Institute Technical Review on the Role of Elastography in Chronic Liver Diseases. Gastroenterology 152:1544–1577 28442120 10.1053/j.gastro.2017.03.016

[14] Augustin S, Millán L, González A et al (2014) Detection of early portal hypertension with routine data and liver stiffness in patients with asymptomatic liver disease: a prospective study. J Hepatol 60:561–569 24211744 10.1016/j.jhep.2013.10.027

[15] Ravaioli F, Colecchia A, Dajti E et al (2018) Spleen stiffness mirrors changes in portal hypertension after successful interferon-free therapy in chronic-hepatitis C virus patients. World J Hepatol 10:731–742 30386466 10.4254/wjh.v10.i10.731 PMC6206152

[16] Murata Y, Abe M, Furukawa S et al (2006) Clinical features of symptomatic primary biliary cirrhosis initially complicated with esophageal varices. J Gastroenterol 41:1220–1226 17287902 10.1007/s00535-006-1914-y

[17] Leung JC, Loong TC, Pang J, Wei JL, Wong VW (2018) Invasive and non-invasive assessment of portal hypertension. Hepatol Int 12:44–55 28361299 10.1007/s12072-017-9795-0

[18] Burghart L, Halilbasic E, Schwabl P et al (2022) Distinct prognostic value of different portal hypertension-associated features in patients with primary biliary cholangitis. J Gastroenterol 57:99–110 34893924 10.1007/s00535-021-01839-3 PMC8831368

